# Multiple Causes of Fatigue during Shortening Contractions in Rat Slow Twitch Skeletal Muscle

**DOI:** 10.1371/journal.pone.0071700

**Published:** 2013-08-16

**Authors:** Kristin Halvorsen Hortemo, Morten Munkvik, Per Kristian Lunde, Ole M. Sejersted

**Affiliations:** 1 Institute for Experimental Medical Research, Oslo University Hospital and University of Oslo, Oslo, Norway; 2 KG Jebsen Cardiac Research Centre and Centre for Heart Failure Research, University of Oslo, Oslo, Norway; Scuola Superiore Sant'Anna, Italy

## Abstract

Fatigue in muscles that shorten might have other causes than fatigue during isometric contractions, since both cross-bridge cycling and energy demand are different in the two exercise modes. While isometric contractions are extensively studied, the causes of fatigue in shortening contractions are poorly mapped. Here, we investigate fatigue mechanisms during shortening contractions in slow twitch skeletal muscle in near physiological conditions. Fatigue was induced in rat soleus muscles with maintained blood supply by *in situ* shortening contractions at 37°C. Muscles were stimulated repeatedly (1 s on/off at 30 Hz) for 15 min against a constant load, allowing the muscle to shorten and perform work. Fatigue and subsequent recovery was examined at 20 s, 100 s and 15 min exercise. The effects of prior exercise were investigated in a second exercise bout. Fatigue developed in three distinct phases. During the first 20 s the regulatory protein Myosin Light Chain-2 (slow isoform, MLC-2s) was rapidly dephosphorylated in parallel with reduced rate of force development and reduced shortening. In the second phase there was degradation of high-energy phosphates and accumulation of lactate, and these changes were related to slowing of muscle relengthening and relaxation, culminating at 100 s exercise. Slowing of relaxation was also associated with increased leak of calcium from the SR. During the third phase of exercise there was restoration of high-energy phosphates and elimination of lactate, and the slowing of relaxation disappeared, whereas dephosphorylation of MLC-2s and reduced shortening prevailed. Prior exercise improved relaxation parameters in a subsequent exercise bout, and we propose that this effect is a result of less accumulation of lactate due to more rapid onset of oxidative metabolism. The correlation between dephosphorylation of MLC-2s and reduced shortening was confirmed in various experimental settings, and we suggest MLC-2s as an important regulator of muscle shortening.

## Introduction

Skeletal muscle contraction is a complex process, and the precise mechanisms that mediate fatigue have yet to be fully understood (for review see [1]). In the present study we used an *in situ* model of dynamic muscle activity [2] which provides an experimental setting with maintained blood supply, and allows an extended investigation of fatigue mechanisms during shortening muscle contractions. The effect of temperature on fatigue development is widely accepted, and the *in situ* model enables fatigue experiments to be performed at physiological temperature (37°C).

Fatigue in skeletal muscles typically develops during repeated shortening contractions, as during walking or running. However, most experimental fatigue studies have analysed isometric muscle contractions, were the muscle generates force at a constant length. A few studies have investigated the effect of contraction mode on fatigue development, and they reported significantly larger fatigue development during shortening (isotonic, dynamic) as compared to isometric contractions [3]–[6], suggesting that fatigue development is dependent on the type of muscle contraction. Hence, results based on isometric contractions may not be sufficient to explain the fatigue development in muscles that shorten. On the myofilament level, the reaction velocity of the cross-bridge cycling (attachment, force development, detachment) is higher during shortening contractions as compared to isometric contractions [7]. During isometric contractions, the high force generating state of the actin myosin interaction is considered the dominant state (after release of inorganic phosphate (P_i_)) [8], and the release of P_i_ is thought to be the rate limiting step of the myofibrillar ATPase cycle [9]. During isotonic shortening, however, only 5% of cycle time is spent in the high force generating configuration [8], and the rate limiting step might be different. Furthermore, using force as the only fatigue variable may underestimate the functional impairment of the fatigued muscle during shortening contractions [2], [5], and additional parameters like extent of muscle shortening and velocities of contraction and relengthening could provide a more comprehensive fatigue analysis.

We have previously reported dephosphorylation of Myosin Light Chain 2 (slow isoform, MLC-2s) during shortening contractions in slow twitch muscle [2], [10]. Such a role of the MLC-2s isoform in slow twitch muscle is in sharp contrast to the role of the MLC-2f isoform in fast twitch muscle, since for the latter several studies have shown that muscle activity leads to hyper-phosphorylation and post-tetanic twitch potentiation (for review see [11]). In the present study, we aimed to further elucidate the role of MLC-2s in slow twitch muscle based on the previous studies from our laboratory. Dephosphorylation of MLC-2 is reported to decrease the calcium (Ca^2+^) sensitivity of myofilaments [12], and we hypothesised that dephosphorylation of MLC-2s contributes to the fatigue-induced reduction in maximal shortening (S_max_) during shortening contractions in slow twitch skeletal muscle.

Although a focus on MLC-2, also other determinants of fatigue were accessed to obtain a comprehensive understanding of fatigue mechanisms during shortening contractions. Intracellular Ca^2+^ initiates the skeletal muscle contraction, and the concentration of cytosolic Ca^2+^ is tightly regulated. In isometric contractions, impaired Ca^2+^ release from the sarcoplasmic reticulum (SR) has been recognized as a contributor to fatigue in isolated muscles, but the exact cause has not been established (for review see [13]). Also, muscle activity has been reported to cause phosphorylation of the ryanodine receptor (RyR) through sympathetic signalling involving protein kinase A [14], [15] which could increase the Ca^2+^ release favourable for contraction, but also increase the SR Ca^2+^ leak and result in slowing of muscle relaxation. Again others have reported reduced SR Ca^2+^ uptake as a contributor to fatigue development in dynamic exercise [16]. Based on the previously observed slowing of relaxation at 100 s exercise [2], we hypothesized that increased SR Ca^2+^ leak and slowing of SR Ca^2+^ uptake contribute to fatigue during shortening contractions.

Recovery from fatigue can provide important information in unfolding the mechanisms of muscle function, and in the present study we followed recovery after 20 s, 100 s and 15 min exercise. Further, subsequent to the recovery from 15 min exercise, a 2^nd^ exercise bout was performed. Earlier work in our group [2] suggested an improved performance following recovery from 15 min of shortening contractions, and we therefore aimed to further describe the contractile function in a subsequent, second (2^nd^) exercise bout and to compare the fatigue mechanisms with those of the prior (1^st^) exercise bout. Prior exercise has been reported to improve several biochemical and physiological effects during a subsequent bout of exercise, possibly due to a more rapid activation of oxidative metabolism [17]–[19]. Munkvik *et al.* [2] previously reported normal tissue oxygen levels at the crucial 100 s time point in the 1^st^ bout of the *in situ* exercise model, suggesting that the rate limiting step is probably downstream of oxygen delivery to the cells.

The aim of this study was to seek an integrative understanding of fatigue mechanisms at play during exercise involving repeated shortening contractions. The *in situ* model of Munkvik *et al* [2] was expanded with additional time points, analyses, recovery tracings, vesicle studies and a comparison with a subsequent exercise bout. We hypothesized that in slow twitch skeletal muscle the regulatory protein MLC-2s affects capacity to shorten.

## Methods

### Ethics Statement

All experiments and animals were handled in strict accordance to the Norwegian Animal Welfare Act, and protocols were approved by the Norwegian Animal Research Authority (approval ID 527). Male Wistar rats (Wistar Hannover, Taconic, Skensved, Denmark) were caged for one week after arrival before included in the study, housed in a controlled environment (temperature 22±2°C, humidity 55±5%, 12/12 hr daylight/night cycle). The animals were fed standard rat chow (B & K Universal, Oslo, Norway) and water ad libitum. In total 115 animals were used.

### Surgery

The rats were anaesthetized in a chamber with 65% N_2_O, 30% O_2_ and ≈5% isoflurane (Forene®), intubated and placed on a respirator (Zoovent, Triumph Technical Services LTD, London, UK) and all efforts were made to minimize suffering. A pressure sensitive catheter (Cardiovascular catheter SPR-407, Millar Instruments, Houston, TX, USA) was inserted through the right carotid artery and positioned in the aortic arch, and blood pressure was continuously measured during the experiment. Isoflurane can be cardiodepressive, and the concentration was carefully adjusted (about 2–3%) to maintain a stable blood pressure. The rats were kept on a heated (37°C) table during the experiment. At the end of the experiment, animals were killed by neck dislocation while still anaesthetized.

Experiments were performed on *in situ* soleus muscle (SOL). The right leg was skinned from the knee and down, and the soleus muscle was dissected free from surrounding tissue except from the blood supply that was kept intact. Great care was taken not to damage or put any drag on the muscle’s blood supply. A surgical thread was placed on the distal tendon just proximal to the calcaneus bone, and the distal attachment of the soleus muscle was released by cutting off a small portion of calcaneus. The tread was tied to the lever arm of a combined force and length transducer (model 305B, Aurora Scientific, Ontario, Canada), placed so that the soleus muscle was parallel to tibia ensuring physiological movement of the muscle. The ankle and the middle part of the tibial bone were clamped, leaving the leg immobile and stable. The sciatic nerve was identified and cut. The soleus’ core temperature was kept at 37°C by preheated 0.9% NaCl constantly running over the epimysium of the muscle.

### Stimulation Protocols

Platinum electrodes were placed at the proximal and distal end of the soleus muscle. The muscle was stretched to ideal sarcomere length (typically representing 3% of maximal force, preload) and stimulated (Pulsar 6 bp, FHC Brunswick, ME, USA) isometrically and supramaximally (8 V) to obtain maximal force at 1 Hz, and at 100 Hz (F_max_).

**Figure 1:**
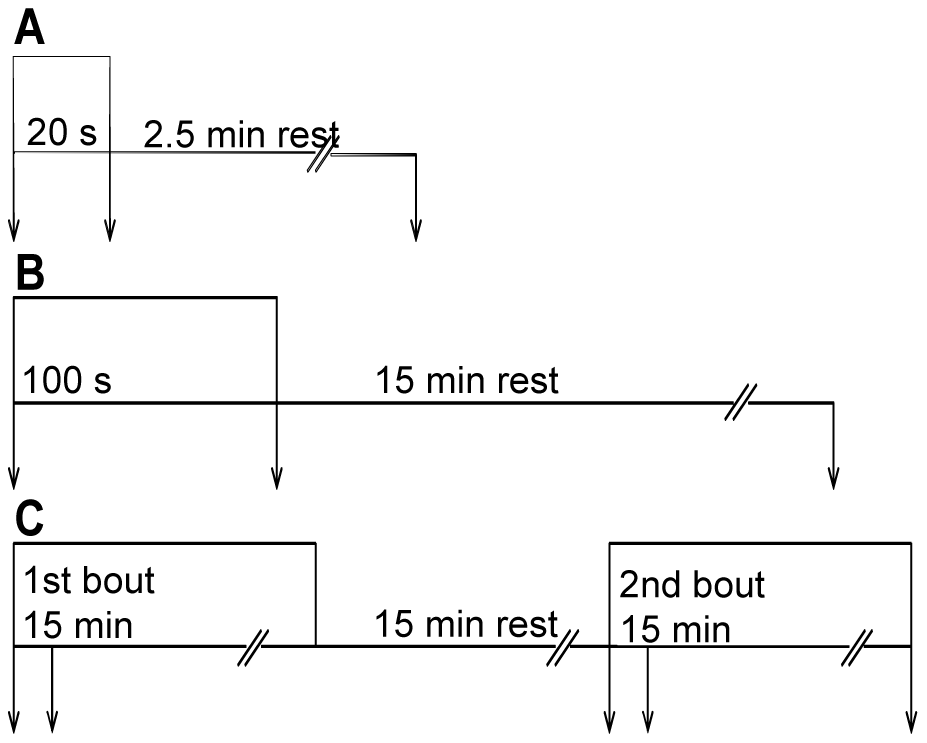
Overview of the three experimental protocols. Intermittent stimulation at 30 Hz for 1 s every 2 s. *A*) 20 s exercise (stimulation) followed by 2.5 min rest. *B*) 100 s exercise followed by 15 min rest. *C*) 15 min exercise (1^st^ bout) followed by 15 min rest before initiating another 15 min exercise (2^nd^ bout). In all protocols, muscles were harvested (arrows) at start and at end of exercise, as well as after rest. In *C*, there was an additional harvesting point at 100 s both in the 1^st^ and 2^nd^ bout.

In the exercise protocols the muscle was allowed to shorten after reaching a pre-set load (afterload). The afterload was set to 33% of F_max_ for that individual muscle. At this afterload muscles are able to produce maximum power, and fatigue most rapidly [5]. The muscle was stimulated intermittently with stimulation trains (1 s on/1 s off) at 30 Hz for 20 s, 100 s or 15 min, with 1 ms pulse duration. Subsequent to the exercise protocols, recovery from fatigue was followed during a 2.5 min resting period (after 20 s exercise) or 15 min resting period (after 100 s and 15 min exercise) (Fig. 1A–C). Recovery of contractile parameters was measured by initiating single stimulation trains at certain intervals during recovery (after 1 and 2.5 min after 20 s exercise and after 1, 2.5, 5, 7.5, 10 and 15 min after 100 s exercise). In regard to the 15 min exercise protocol (1^st^ bout), another 15 min of shortening contractions (2^nd^ bout) was initiated after the 15 min rest period (Fig. 1C) for comparison with the 1^st^ bout.

To investigate the potential effect of afterload on MLC-2s phosphorylation, some additional 15 min exercise protocols were performed with the afterload altered to 10, 20, 33 or 100% of F_max_. Similarly, the effect of stimulation frequency on MLC-2s phosphorylation was investigated by keeping a constant afterload (33% of F_max_) while stimulation frequency was set to 20, 30 or 40 Hz. Maximal shortening and force development were recorded at the various frequencies before initiating the exercise protocols.

Blood pressure, superficial muscle temperature, stimulation pulse, force and shortening were displayed and sampled at 2000 Hz by a custom-made LABview program. Subsequent curve fittings were done by the use of TableCurve 2D (v5.0 Systat Software, Chicago, IL, USA) or SigmaPlot (Systat Software Inc, version 10.0) and calculations of contractile parameters were done with Microsoft Excel 2007. Definitions of analysed contractile parameters are presented in Table 1.

**Table 1:** Definitions of calculated contractile parameters.

	Definition (unit)
T_bl_	Baseline tension (mN)
F_max_	Maximal isometric tetanic force (N)
dF/dt	Maximal rate of isometric force development (N s^-1^)
-dF/dt	Maximal isometric relaxation rate (N s^-1^)
dL/dt	Maximal isotonic shortening velocity (mm s^-1^)
-dL/dt	Maximal isotonic relengthening velocity (mm s^-1^)
L_0_	Optimum resting length (mm)
S_max_	Maximal shortening (mm)
TTL_0_	Time to optimum resting length (ms)
tau1	Time constant of the rapid component of isometric relaxation (ms)
tau2	Time constant of the slow component of isometric relaxation (ms)

### Metabolites

Muscles were harvested and frozen in liquid nitrogen (within 5 sec after blood supply was cut) at the appropriate exercise and recovery times, and stored at −80°C. The contralateral (resting) soleus muscle served as control. Extraction of metabolites was done from frozen muscle tissue by incubation in ice cold 3 M perchloric acid (PCA; HClO_4_) and neutralizing with KHCO_3_. The muscle content of CrP, ATP, ADP and AMP was determined by HPLC, and muscle lactate content was determined by a fluorometric enzymatic coupled assay [20]. Munkvik *et al.* [2] reported normal tissue oxygen levels at both 100 s and 15 min exercise in the *in situ* protocol, and the measurements were not repeated in this study.

### Myosin Light Chain 2 (MLC-2) Analysis

Myofibrillar proteins were isolated and subsequently separated by SDS polyacrylamide gel electrophoresis. Phosphorylation level was analysed by sequentially staining the gels with fluorescent stains which is specific for phosphorylated proteins (ProQ Diamond) and total proteins (SYPRO Ruby) (both Molecular Probes, Invitrogen, Oslo, Norway). Bands were detected by Typhoon laser scanner (Typhoon 9410, GE Healthcare, Oslo, Norway), and staining intensity was quantified by ImageQuant (GE Healthcare, Oslo, Norway). Phosphorylation level was calculated in individual muscles by dividing the staining intensity reflecting phosphorylation level (ProQ Diamond) by the staining intensity reflecting the protein content (subsequent staining of the same gel with Sypro Ruby) of the MLC-2s protein band. Further, this normalized phosphorylation level of MLC-2s in the exercised muscle was divided by the normalized phosphorylation level of MLC-2s in the contralateral resting control muscle, and presented as MLC-2s phosphorylation relative to the resting control (%).

Parallell to the ProQ Diamond/Sypro Ruby gel staining, samples were also analysed by Western blot which verified the MLC-2s band (anti-MLC-2 (F109.3E1), BioCytex). Western blot with a phosphorylation specific antibody (anti-MLC-2 pSer18 (AP08007PU-N), Nordic BioSite) was performed on a selection of samples to verify the pattern of MLC-2s phosphorylation observed with ProQ Diamond/Sypro Ruby gel stain. In the Western blot analyses, loading control was performed by Coomassie staining for total protein content.

### SR Calcium uptake, Release and Leak

Sarcoplasmic reticulum (SR) function was measured in SR vesicles from whole muscle homogenates, based on methods described previously [21]–[23]. Soleus muscles from exercised leg were quickly excised, and approximately 40 mg tissue was homogenized in ice cold buffer (300 mM sucrose, 1 mM EDTA, 5 mM NaN_3_, 40 mM Tris HCl, 40 mM L-histidine, pH 7.9) before frozen in liquid nitrogen and stored at −80°C. The contralateral resting leg served as control.

LS50B spectrophotometer (Perkin Elmer Ltd, Beaconsfield, Buckinghamshire, UK) was used to analyse SR function at 37°C by measuring the intensities of the calcium binding dye fura-2. Fura-2 (final 0.8 µM) and protein homogenate (final protein concentration ≈ 1 mg mL^-1^), was added to the assay buffer (165 mM KCl, 22 mM Hepes, 7.5 mM oxalate, 11 mM NaN_3_, 5.5 µM TPEN, 4.5 mM MgCl_2_, 100 mM Tris HCl, pH 7.0), subsequently SR Ca^2+^ uptake was initiated by adding MgATP (final 1 mM) and blocked by thapsigargin (final 1.5 µM). SR leak was reported as the linear rate of rise in Ca^2+^ during the following 60 s after addition of thapsigargin. Addition of 4-Chloro-m-Cresol (final 10 mM) initiated Ca^2+^ release from SR.

Each Ca^2+^ fluorescence curve was smoothed using the Savitzky-Golay algorithm (TableCurve 2D v5.0, Systat Software, Chicago, IL, USA), and converted to free [Ca^2+^] according to the equation: [Ca^2+^]_free_ = K_d_[(R-R_min_)/(R_max_-R)](S_f2_/S_b2_) using Kd224 nM for fura-2 [24]. R_min_ and R_max_ is the ratio when fura-2 is free from (EGTA, final 3.2 mM) or saturated with Ca^2+^ (CaCl_2_, final 4.8 mM), respectively, whereas S_f2_/S_b2_ is the corresponding ratios in fluorescence intensity. The R_min_, R_max_ and S_f2_/S_b2_ were determined in each run. The Ca^2+^ uptake rate was obtained as the first derivative of the smoothed [Ca^2+^]_free_ versus time curve. The rates were corrected for total muscle protein (µmol s^-1^ mg protein^-1^). The [Ca^2+^]_free_ *vs.* Ca^2+^ uptake rate were fitted to a Hill equation (SigmaPlot 11.0, Systat Software, Inc. San Jose, CA, USA) to obtain values for the maximal Ca^2+^ uptake rates (V_max_). The maximum Ca^2+^ release rate was calculated by fitting the release data to an exponential function ([Ca^2+^]_free_ = a(1−e^τt^)).

### Statistics

Data are expressed as means ± SEM if not otherwise specified. For all tests, p<0.05 was considered significant. Differences between two groups were tested using Student’s paired- or unpaired t-test, Welch’s t-test or Mann-Whitney Rank Sum Test whenever appropriate. One-way ANOVA was used for multiple comparisons *vs.* a control group, and one-way repeated measures ANOVA was used for repeated measures. The statistical analyses were performed by means of Statistica (version 8.0, StatSoft, Tulsa, OK), SigmaPlot (Systat Software Inc, version 10.0) or Microsoft Excel 2007.

## Results

### Animal Characteristics

The weight of animals was 334±4 g (n115) at the time of experiment. There was little variability in twitch tension (0.6±0.01 N) and F_max_ (2.1±0.03 N), hence the set afterload (33% of F_max_) was not different between animals.

### Outline of the Exercise Protocols

**Figure 2:**
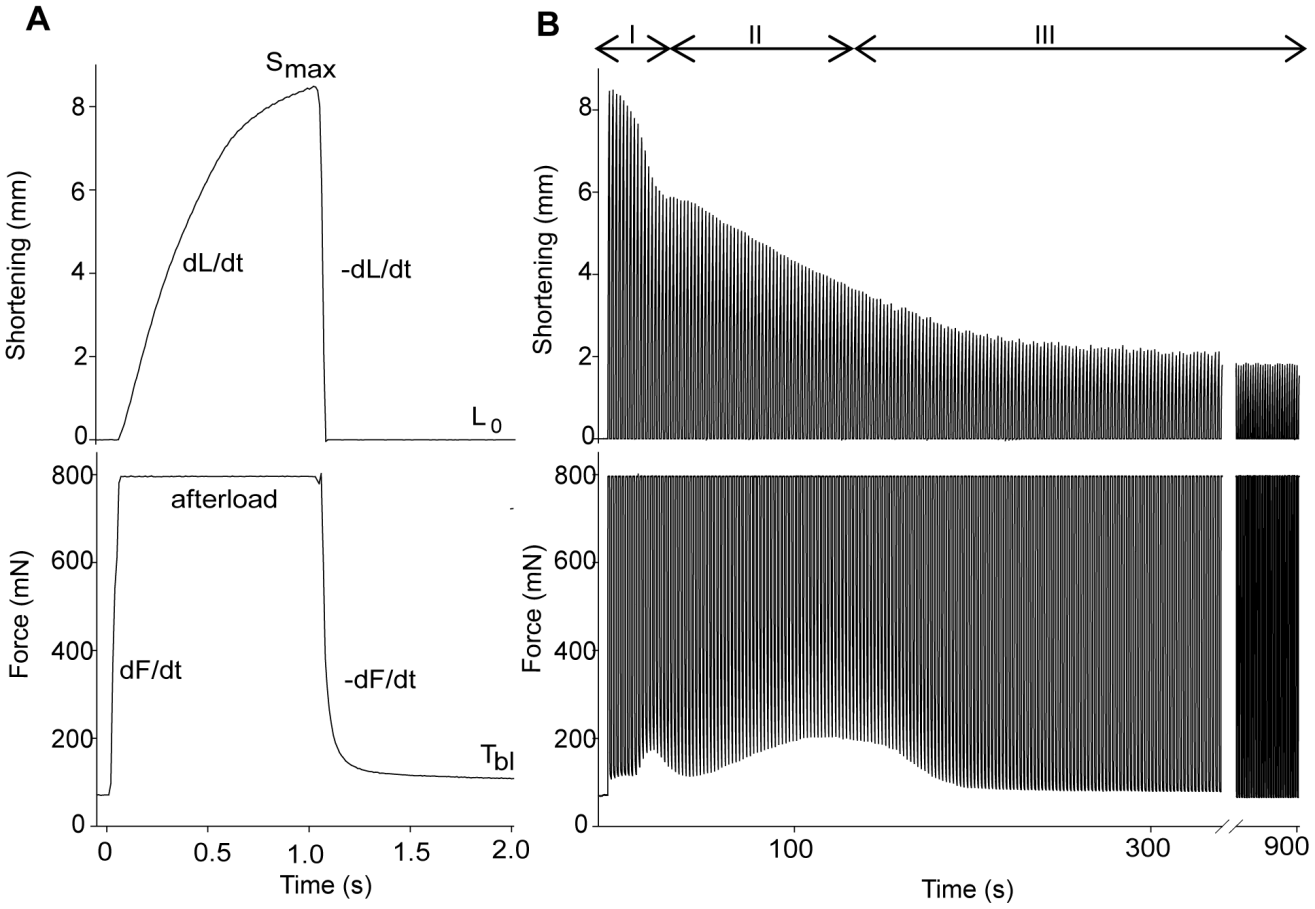
Representative tracings of shortening and force in the 15 min exercise protocol (1^st^ bout). Tracings of shortening (upper panel) and force (lower panel). *A*) The *in vivo* prepared muscle was stimulated intermittently with 1s on, 1s off. As stimulation starts, force rises from resting base line tension (T_bl_) until it reaches the pre-set afterload (33% of F_max_). From here, the force is maintained while the muscle starts to shorten and reaches the maximal shortening (S_max_). When stimulation ceases (after 1 s) the muscle is passively stretched to resting length (L_0_), and subsequently force declines. *B*) Representative tracing of a 15 min exercise protocol (1^st^ bout). Note the three phases marked with roman numbers; during the first 20 s exercise there is a prominent fall in S_max_ (upper panel); at 100 s there is a further reduction in S_max_ accompanied by a transient rise in T_bl_ (lower panel); at 15 min there is still reduction in S_max_ while T_bl_ is restored to initial values.

Each stimulation train (30 Hz, 1 s) evoked a rapid rise in force (isometric force development) until the set afterload was reached (i.e. 33% of F_max_), subsequently the muscle started to shorten (isotonic shortening) (Fig. 2A). After 1 s, the stimulation ceased and the muscle was relengthened to resting length (isotonic relengthening), followed by a decline in tension (isometric relaxation). The base line tension (T_bl_) (Fig. 2A) recorded just before the next stimulation train was influenced by the changing relengthening and relaxation rates. The total time available for relengthening and relaxation was 1 s between each stimulation train.

Fatigue is dependent on contraction mode, work intensity and duration of activity. Importantly, fatigue defined as the progressive loss of force generating capacity or decline in F_max_ [25] will not reflect the reduction in muscle performance observed during shortening contractions; at 15 min of strenuous shortening contractions there was no significant reduction in F_max_ [2]. Since we used the same protocol as in [2], we did not measure F_max_ during the experiment (but F_max_ was measured before the exercise protocol and used to calculate afterload). To describe fatigue we assessed in detail the rates of isometric force development and relaxation, the velocities of isotonic shortening and relengthening as well as the resulting S_max_ and T_bl_. We identified three distinct time phases during the exercise protocol (Fig. 2B): the first phase from start to 20 s (rapid decline of S_max_), the second phase from 20 s to 100 s (increase of T_bl_ and further fall of S_max_), and the third phase from 100 s to 15 min (normalization of T_bl_ and sustained low S_max_).

Recovery from fatigue was followed after 20 s, 100 s and 15 min of exercise. Earlier work in our group reported an improved performance following recovery from 15 min of shortening contractions. Therefore, after 15 min rest we also performed a 2^nd^ bout of 15 min exercise for comparison with the 1^st^ bout.

### Fatigue and Recovery

#### Fatigue at 20 s exercise

**Figure 3:**
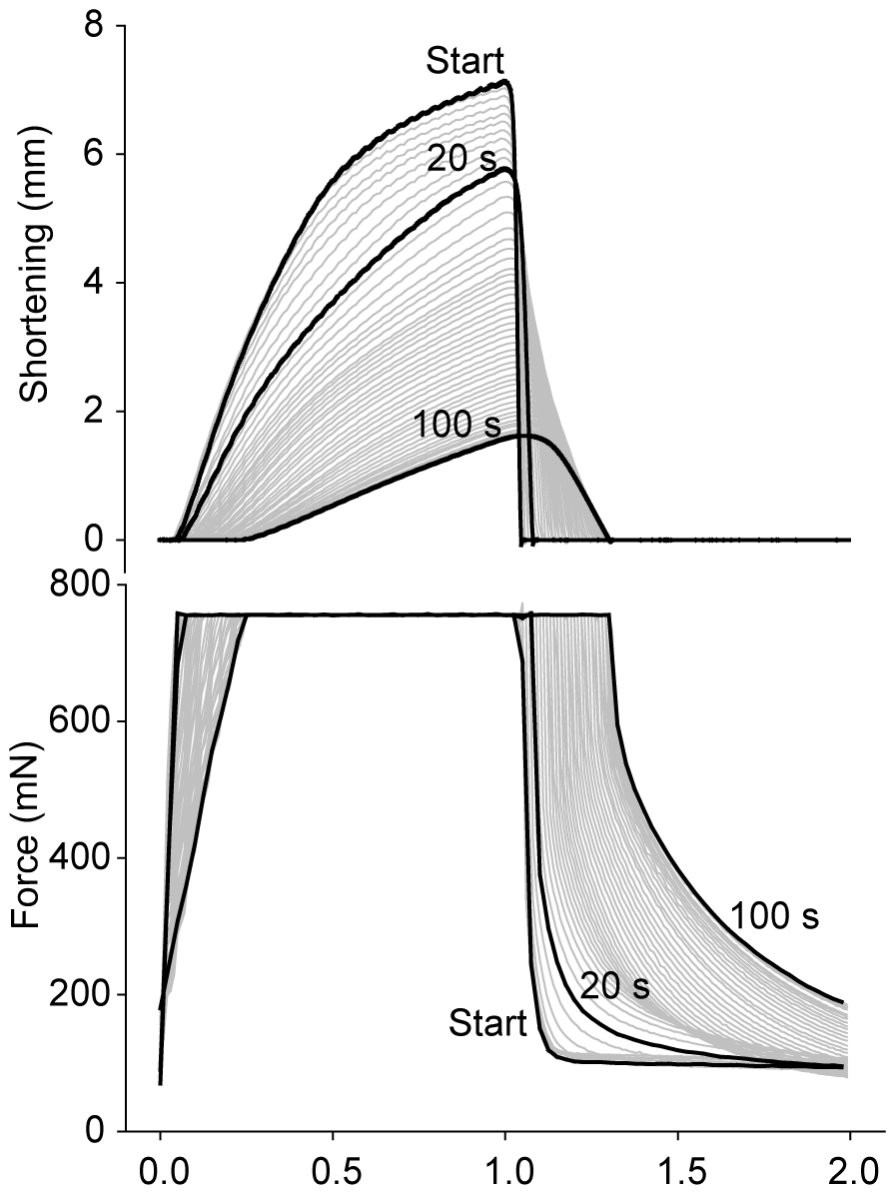
Characteristic fatigue development during the first 100 s of exercise. Representative tracings of shortening (upper panel) and force (lower panel) assembled from the first 100 s. Tracings at start, 20 s and 100 s exercise are highlighted (black). Note the reduced shortening already at 20 s exercise. At 100 s exercise, there is a further reduction in shortening, and there is also a slowing of the remaining phases of the contraction cycle and especially a significant prolongation of the isometric relaxation phase (lower panel).

**Figure 4:**
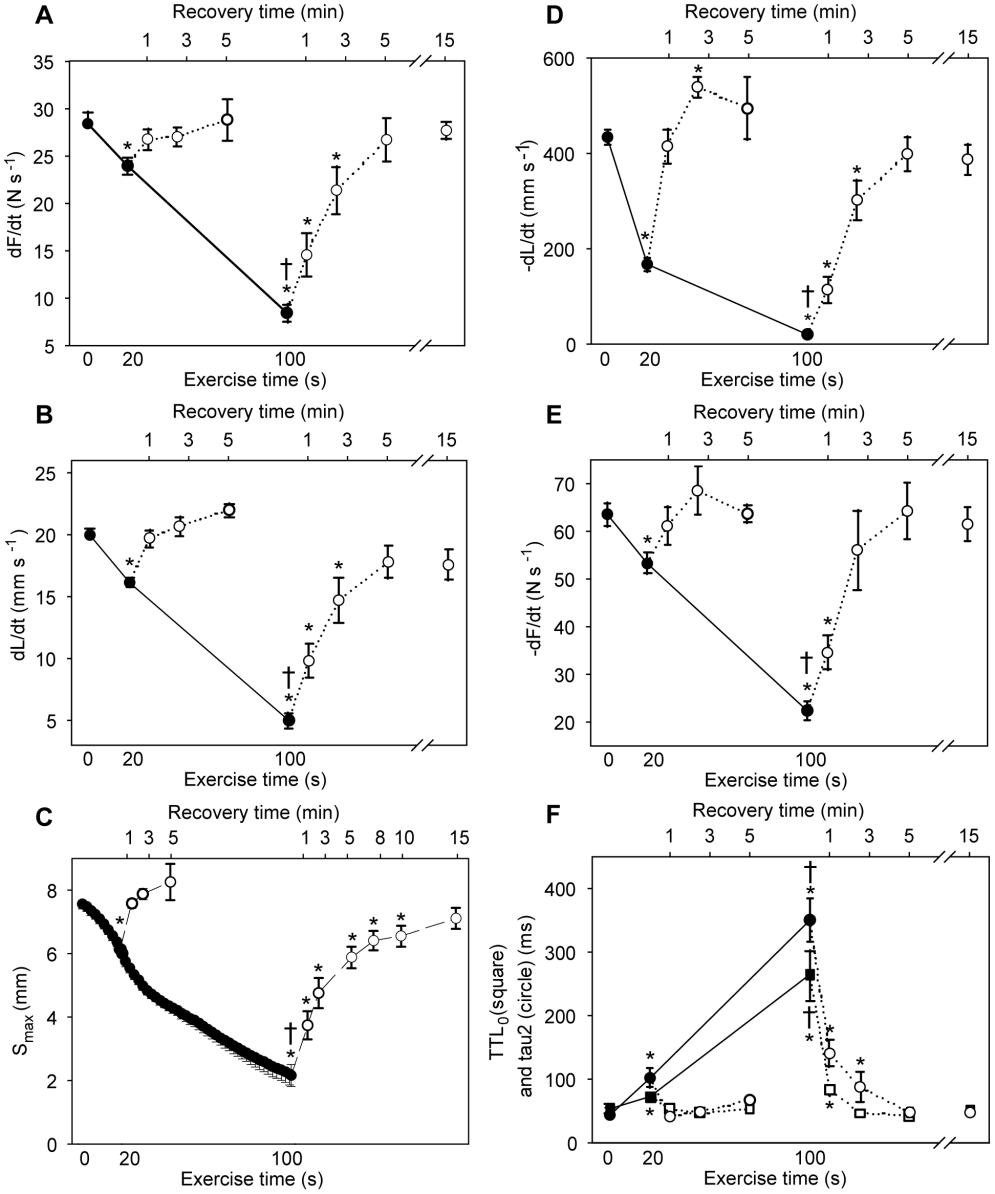
Time course of fatigue and recovery during 20 s and 100 s exercise. The panels show development of contraction (panels *A*, *B* and *C*) and relaxation (panels *D*, *E* and *F*) parameters during fatigue development (black symbols, solid line) and recovery (white symbols, dotted line). Exercise times are given at bottom x-axis and recovery times at top x-axis. *A)* Maximal rate of isometric force development, dF/dt. *B*) Maximal isotonic shortening velocity, dL/dt. C) Maximal shortening, S_max_. D) Maximal isotonic relengthening velocity, −dL/dt. E) Maximal isometric relaxation rate, −dF/dt. *F*) Time to resting length, TTL_0_ (squares) and tau2 (circles). Symbols are averages ± SEM. * p<0.05 *vs.* initial value. † p<0.05 *vs.* 20 s. *N* start*24*; 20 s = *12*; 100 s = *12*; 20 s +2.5 min recovery = *6*; 100 s +15 min recovery = *6*.

Signs of fatigue were clearly evident already at 20 s of stimulation. The most prominent finding was a reduction of S_max_ to 80% of initial value (p<0.05; Fig. 3) caused by a corresponding reduction in maximal isotonic shortening velocity (dL/dt) (Fig. 4B). This reduction during the first 20 s was significantly more pronounced than the subsequent more gradual decline of S_max_ evident after 100 s and 15 min (Fig. 2B and 4C).

With regard to the other phases of the contraction-relaxation cycle, the maximal rate of isometric force development (dF/dt) was slightly reduced to 89% (p<0.05 compared to initial value) (Fig. 4A). The maximal isotonic relengthening velocity (-dL/dt) was reduced to 40% of initial value at 20 s (Fig. 4D), but the resulting increase in isotonic relengthening time (time to resting length, TTL_0_) was less affected (Fig. 4F), partly due to the corresponding reduction in S_max_. In the last part of the contraction-relaxation cycle, i.e. the isometric relaxation, maximal relaxation rate (-dF/dt) was reduced to 84% of initial value at 20 s (Fig. 4E). The isometric relaxation phase was fitted to a double exponential decay curve, and the two time constants tau1 and tau2 were increased from 4.5±0.4 to 8.4±1.3 ms (p<0.05) and from 41±3 to 140±25 ms (p<0.05, Fig. 4F), respectively. Correspondingly, there was a modest increase in T_bl_ from the initial 70±1 to 122±9 mN (p<0.05).

#### Recovery after 20 s exercise

After cessation of exercise, the muscle was allowed to rest and the contractile properties gradually recovered. The reduction in S_max_ observed at 20 s was rapidly normalized, with recovery to initial values already after 1 min rest (Fig. 4C). The recovery of S_max_ corresponded to the recovery of dL/dt (Fig. 4B). All other measured contractile parameters were also fully recovered after 1 min rest (Fig. 4A–F).

#### Fatigue at 100 s exercise

Muscle function had deteriorated further at 100 s exercise (Fig. 3), with S_max_ reduced to 32% of initial value (p<0.05) (Fig. 4C). In contrast to the 20 s time point, there were prominent signs of fatigue at 100 s also in the other phases of the contraction-relaxation cycle. Especially, there was a marked slowing of the isometric relaxation phase with a characteristic rise in T_bl_.

Compared to initial values the dF/dt was reduced to 29% (p<0.05) (Fig. 4A) and time to reach afterload increased correspondingly (from the initial 41±1 to 153±13 ms, p<0.05). The relengthening phase was also significantly affected at 100 s with –dL/dt only 6% of initial value (Fig. 4D), but the concomitant decline of S_max_ attenuated the effect on TTL_0_, which however was significantly prolonged (Fig. 4F). This prolongation of TTL_0_ resulted in less time available for isometric relaxation before the next stimulation train. There was marked slowing of isometric relaxation; -dF/dt was down to 34% of initial rate (p<0.05) (Fig. 4E) and the time constant tau1 exhibited a fourfold (17±2, p<0.05) and tau2 an almost nine fold prolongation (to 384±53 ms, p<0.05, Fig. 4F) compared to initial values. This increase of tau2 explained most of the characteristic rise in T_bl_, which reached a maximum value at 100 s stimulation (from the initial 70±1 to 219±18 mN, p<0.05).

#### Recovery after 100 s exercise

Recovery following 100 s of exercise took longer compared to recovery following 20 s exercise. This was mainly because the contractile parameters at 100 s were further deteriorated, since initial rates of recovery were about the same (Fig. 4). S_max_ recovered rapidly at first, but was fully normalized only after 15 min rest (Fig. 4C). In contrast, the remaining measured contractile parameters were fully recovered to initial values already after 5 min rest (Fig. 4A–F).

#### Fatigue at 15 min exercise

**Figure 5:**
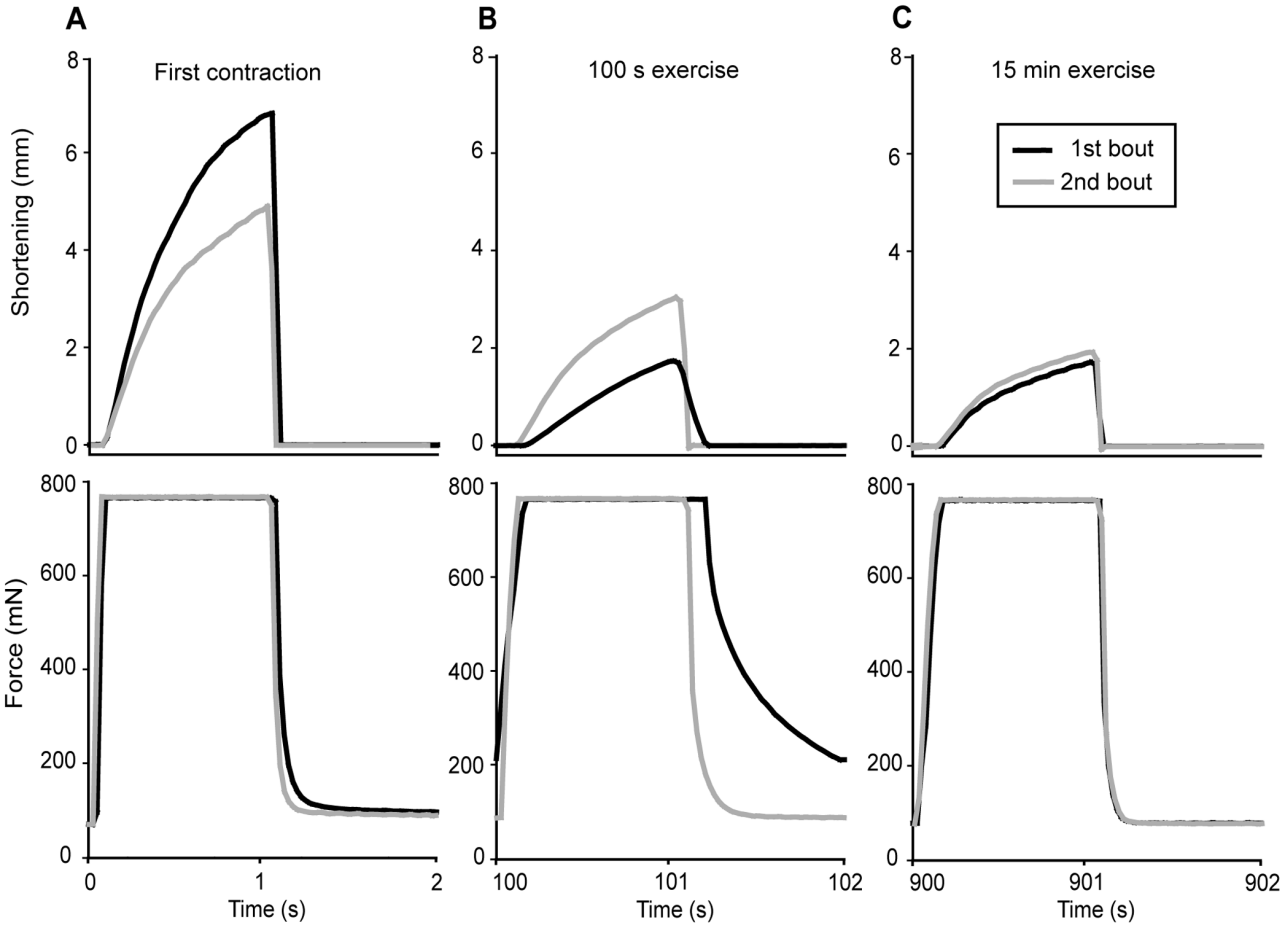
Expanded view of representative tracings at selected time points in the 1^st^ and 2^nd^ bout. Shortening (upper panels) and force (lower panels) from a representative tracing at different time points in the 1^st^ (black) and 2^nd^ (grey) exercise bout. *A*) The initial exercise cycle. *B*) At 100 s of exercise. *C*) At 15 min exercise.

Continued exercise subsequent to the 100 s time point did not aggravate signs of fatigue any further. On the contrary, some of the deteriorated parameters were restored to initial values in the course of continued exercise. However, S_max_ remained suppressed. As illustrated by a typical experiment in Fig. 5, the isometric part (black line, lower panels) of the exercise cycle at 15 min (Fig. 5C) resembled the first contraction cycle (Fig. 5A). In contrast, the isotonic phase (black line, upper panels) of the exercise cycle at 15 min (Fig. 5C) was not very different from the 100 s time point (Fig. 5B).

**Figure 6:**
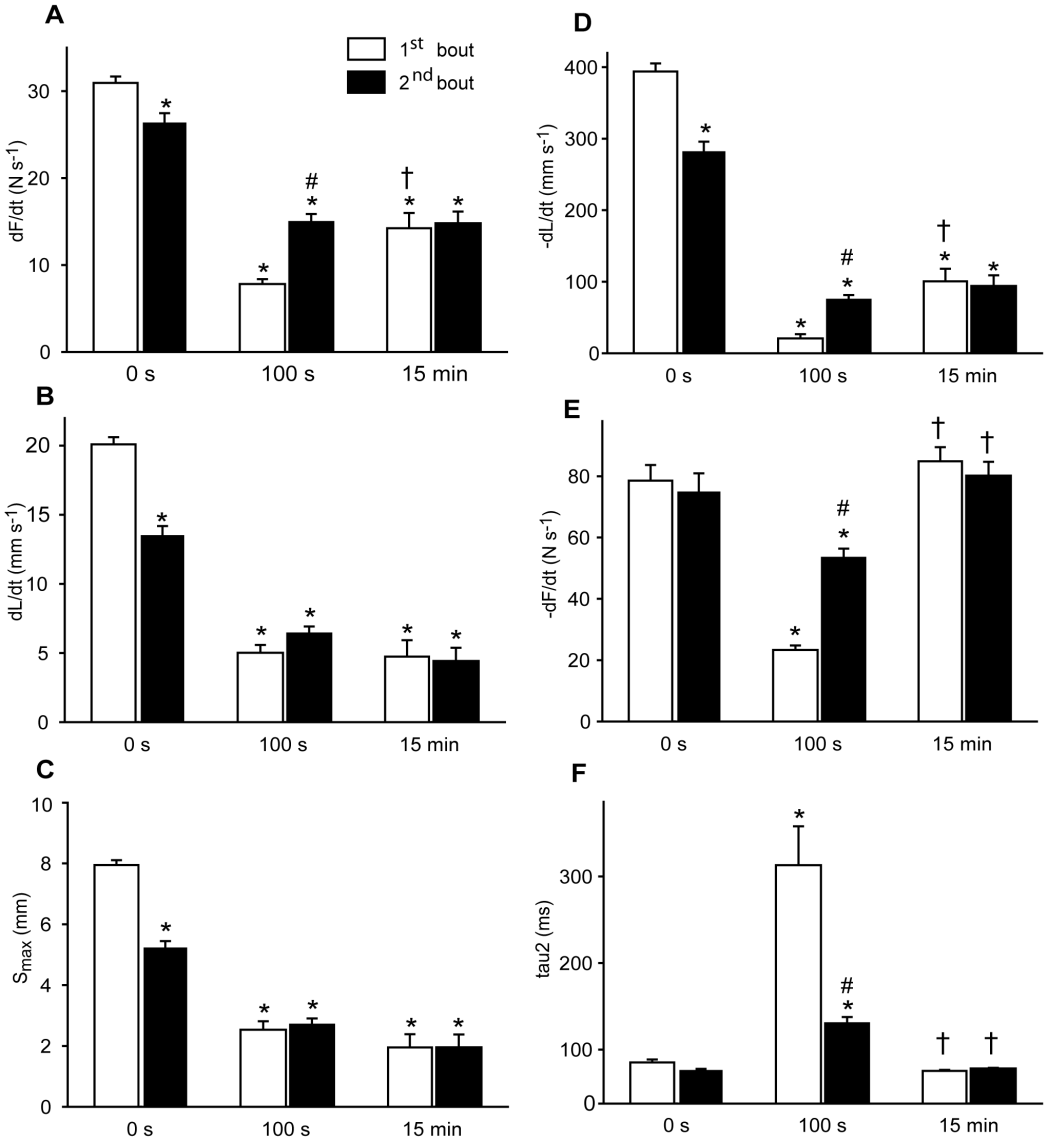
Contractile performance in the 1^st^ bout (open bars) *vs.* the 2^nd^ bout (black bars). Contraction (panels *A*, *B* and *C*) and relaxation (panels *D*, *E* and *F*) parameters at start (0 s), at 100 s and at 900 s of shortening contractions. *A*) Maximal rate of isometric force development, dF/dt. *B*) Maximal isotonic shortening velocity, dL/dt. *C*) Maximal shortening, S_max_. *D*) Maximal isotonic relengthening velocity, −dL/dt. *E*) Maximal isometric relaxation rate, −dF/dt. *F*) Tau2 values. Bars are averages ± SEM. * p<0.05 *vs.* initial value. # p<0.05 *vs.* corresponding 1^st^ bout value. † p<0.05 *vs.* 100 s. *N* start 1^st^ = *20*; 100 s 1^st^ = *12*; 15 min 1^st^ = *6*; start 2^nd^ = *12*; 100 s 2^nd^ = *20*; 15 min 2^nd^ = *6*.

In detail, the dF/dt was partly restored to 45% of initial value at 15 min exercise, significantly improved compared to at 100 s (Fig. 6A), while dL/dt and S_max_ at 15 min were not significantly different from the 100 s time point (Fig. 6B–C). A partial and significant restoration of –dL/dt to 24% of initial value (Fig. 6D) was sufficient to restore the TTL_0_ to initial value (50±2 ms, ns) at 15 min due to the persistent reduction in S_max_. Strikingly, during the course of continued exercise, the isometric relaxation parameters were fully restored at 15 min exercise (ns *vs.* initial values, Fig. 6E–F). As a result, T_bl_ returned to initial value (68±5 mN, ns *vs.* initial value).

#### Recovery after 15 min exercise

The first contraction (0 s) of the 2^nd^ exercise bout corresponds to recovery 15 min after cessation of the 1^st^ exercise bout. A representative tracing of the first contraction cycle in the 2^nd^ bout (grey line) compared to initial performance in the 1^st^ bout (black line) is found in Fig. 5A, displaying partial overall recovery.

In detail, there was almost complete recovery of the isometric contraction phase, with dF/dt recovered to 89% of initial value (p<0.05) (Fig. 6A). There was remarkably less recovery of dL/dt (recovered to 67% of initial value, p<0.05) with a corresponding partial recovery of S_max_ (Fig. 6B-C). In the isotonic relaxation phase there was recovery of -dL/dt (Fig. 6D) enough to produce a TTL_0_ not different from initial values (49±2 ms). The parameters of isometric relaxation were fully restored already at the end of the 1^st^ exercise bout, as described above (*Fatigue at 15 min exercise*), and did not change any further during the 15 min resting period (Fig. 6E-F).

### 2^nd^ Exercise bout

The 2^nd^ exercise bout was performed to examine the effect of a prior fatiguing exercise bout (1^st^ bout) on muscle performance during an identical, subsequent exercise protocol. The comparison was focused on the 100 s time point (which was identified as a highly fatigued state in the 1^st^ bout) as well as the stabilized 15 min time point. Notably, in spite of incomplete recovery of the isotonic contractile parameters at start of 2^nd^ exercise bout, reduction of performance throughout the 2^nd^ bout was less compared to the 1^st^ bout.

#### Fatigue at 100 s exercise in the 2^nd^ bout

A representative tracing of the contractile function at 100 s in the 2^nd^ bout (grey line) compared to the 1^st^ bout (black line) is shown in figure 5B. As in the 1^st^ bout, dL/dt and S_max_ fell during the first 100 s of the 2^nd^ bout, but relatively less so that the absolute value at 100 s was not significantly different from the corresponding 1^st^ bout value (Fig. 6B–C). The other phases of the contraction-relaxation cycle were also better preserved, and interestingly, several parameters were even superior at 100 s in the 2^nd^ bout compared to the corresponding 1^st^ bout values. This was the case for both dF/dt (Fig. 6A) with a correspondingly shorter time to reach afterload (101±7 *vs.* 154±13 ms, p<0.05) and for -dL/dt (Fig. 6D) with a less prolonged TTL_0_ (75±3 ms vs. 184±27 ms, p<0.05). However, the most interesting finding at 100 s in the 2^nd^ bout was evident in the isometric relaxation phase, with significantly less reduced -dF/dt (Fig. 6E) and tau 2 (Fig. 6F) compared to at 100 s in the 1^st^ bout. Tau1 was even fully preserved compared to initial value (5.6±0.8 ms, ns). As a consequence of the less affected relengthening and relaxation rates, the distinctive rise in T_bl_ observed at 100 s in the 1^st^ bout was almost undetectable at 100 s in the 2^nd^ bout (87±2 mN, p<0.05 *vs.* 100 s 1^st^ bout) (exemplified in Fig. 5B, lower panel).

#### Fatigue at 15 min exercise in the 2^nd^ bout

Figure 5C shows a representative tracing of a contraction cycle at 15 min exercise in the 2^nd^ bout (grey line) compared to the 1^st^ bout (black line), illustrating that the contractile functions at 15 min exercise were not different in the two bouts (for details see Fig. 6). As during the 1^st^ bout, the most prominent sign of fatigue at 15 min exercise was a persistent reduction in dL/dt and S_max_, while the remaining contractile parameters were partially or fully restored.

### Metabolites

#### 20 s and 100 s exercise

The high-energy phosphates ATP and CrP were significantly decreased to 70% and 36% of initial value already at 20 s exercise (Table 2) and were further reduced to 52% and 23% of initial values at 100 s, respectively. Muscle lactate was increased about two-fold at 20 s exercise (p<0.05 *vs.* control), and accumulated further to reach a five-fold increase at 100 s exercise (p<0.05 *vs.* 20 s). There was also a rise in AMP at 100 s exercise, while no significant increase in ADP was detected. Metabolite content at 15 min exercise in the 1^st^ bout was investigated in a previous work from our group [2], and was not measured in the present study.

**Table 2:** Metabolites in soleus muscle at rest (Ctr) and at different exercise and recovery times.

	Exercise	*n*	CrP	ATP	ADP	AMP	Lactate
**Ctr**	(0 s)	*52*	10.3±0.5	3.5±0.1	0.69±0.03	0.19±0.01	3.1±0.2
**1^st^ bout**	20 s	*8*	5.1±0.6*	2.7±0.1*	0.61±0.07	0.19±0.03	7.6±0.9*
	100 s	*13*	3.0±0.4* †	2.2±0.2* †	0.82±0.03	0.31±0.04*	17.2±1.0* †
**Recovery**	20 s +2,5 min rec.	*6*	10.7±0.4	2.6±0.3	0.50±0.06	0.14±0.03	6.1±0.9*
	100 s +15 min rec.	*5*	9.3±1.6	2.9±0.4	0.60±0.05	0.16±0.03	6.4±0.8*
	15 min +15 min rec.^1^	*7*	10.0±1.0	2.8±0.1	0.62±0.02	0.14±0.03	6.2±0.5*
**2^nd^ bout**	100 s	*7*	2.4±0.3*	1.5±0.2* #	0.68±0.06	0.24±0.03	10.6±1.3* #
	15 min	7	8.0±1.3	2.8±0.3	0.78±0.07	0.19±0.04	6.4±0.9*

Values are in mmol kg wet weight^-1^, average ± SEM.

* p<0.05 *vs.* control,

† p<0.05 *vs.* 20 s exercise,

# p<0.05 *vs*. 100 s exercise in the 1^st^ bout. *Rec*. recovery.

1 i.e. at start of the 2^nd^ bout.

#### Recovery from 20 s, 100 s and 15 min exercise

Following 20 s exercise, metabolite content was analysed after 2.5 min rest, and following 100 s and 15 min exercise, metabolite content was analysed after 15 min rest (Table 2). During all protocols, muscle metabolite content was fully recovered after the resting period with the exception of muscle lactate that stabilized at a partially recovered level.

#### 2^nd^ bout

In the 2^nd^ bout, metabolites were measured at start (i.e. recovery after 15 min exercise) and at 100 s and 15 min exercise (Table 2). The metabolite content at start of the 2^nd^ bout was not different from at start of the 1^st^ bout with the exception of the not fully recovered muscle lactate. However, at 100 s in the 2^nd^ bout, lactate accumulation was significantly less than the corresponding 1^st^ bout value (10.60 *vs.* 17.21, p<0.001). At 15 min exercise in the 2^nd^ bout, metabolite content was fully restored with the exception of lactate that was partially restored and not different from at start of the 2^nd^ bout.

#### Correlates of contractile function and metabolites

**Figure 7:**
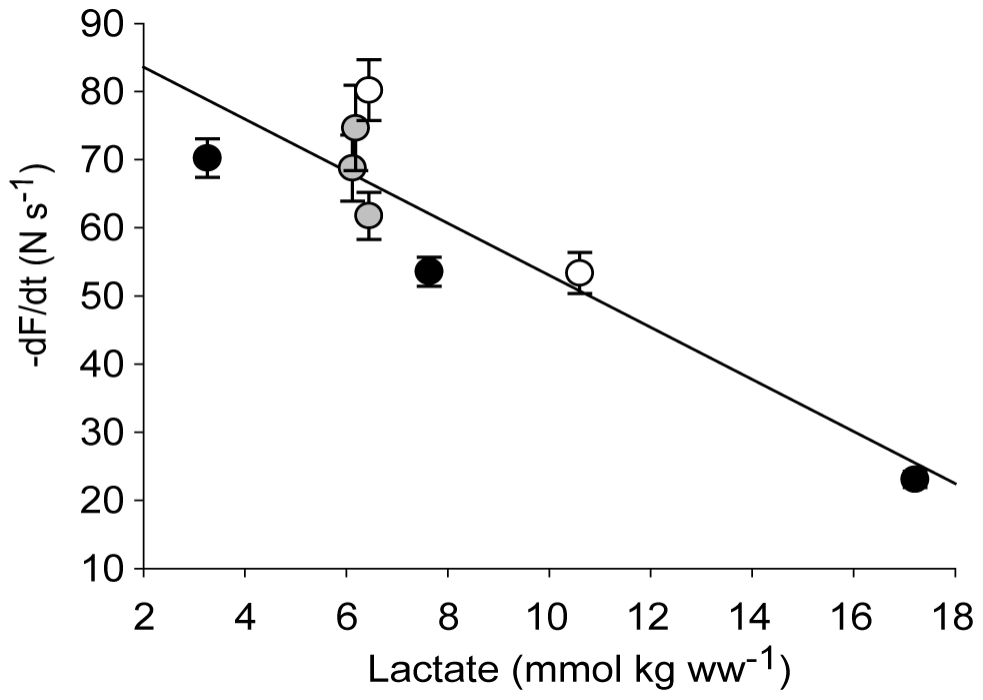
Isometric relaxation and lactate. Isometric relaxation rate (−dF/dt) was strongly correlated to muscle lactate throughout the exercise protocols. Data are obtained from all measured time points in the 1^st^ bout (black), after recovery (grey) and in the 2^nd^ bout (white), presented as a linear regression (r^2^ = 0.81, p<0,01) based on group means ± SEM.

In the 1^st^ bout, the contractile parameters deteriorated in parallel with reduction in CrP and ATP and accumulation of lactate. Further, the complete recovery of contractile parameters following the resting period after both 20 s and 100 s exercise was accompanied by fully recovered muscle metabolite content (except for the partial recovery of lactate). We found that there was an overall correlation between slowing of –dF/dt and accumulation of lactate, also when including measurements from the 2^nd^ bout (Fig. 7). In contrast, the correlation between S_max_ and metabolites weakened when including the 2^nd^ bout values; while in the 1^st^ bout the recovery of metabolites was accompanied by a fully recovered S_max_ (after recovery from 20 s and 100 s exercise), S_max_ at start of the 2^nd^ bout was reduced (Fig. 6C) despite fully recovered metabolites. Hence, metabolites alone could not provide an explanation for the reduced shortening capacity.

### Dephosphorylation of Myosin Light Chain 2

**Figure 8:**
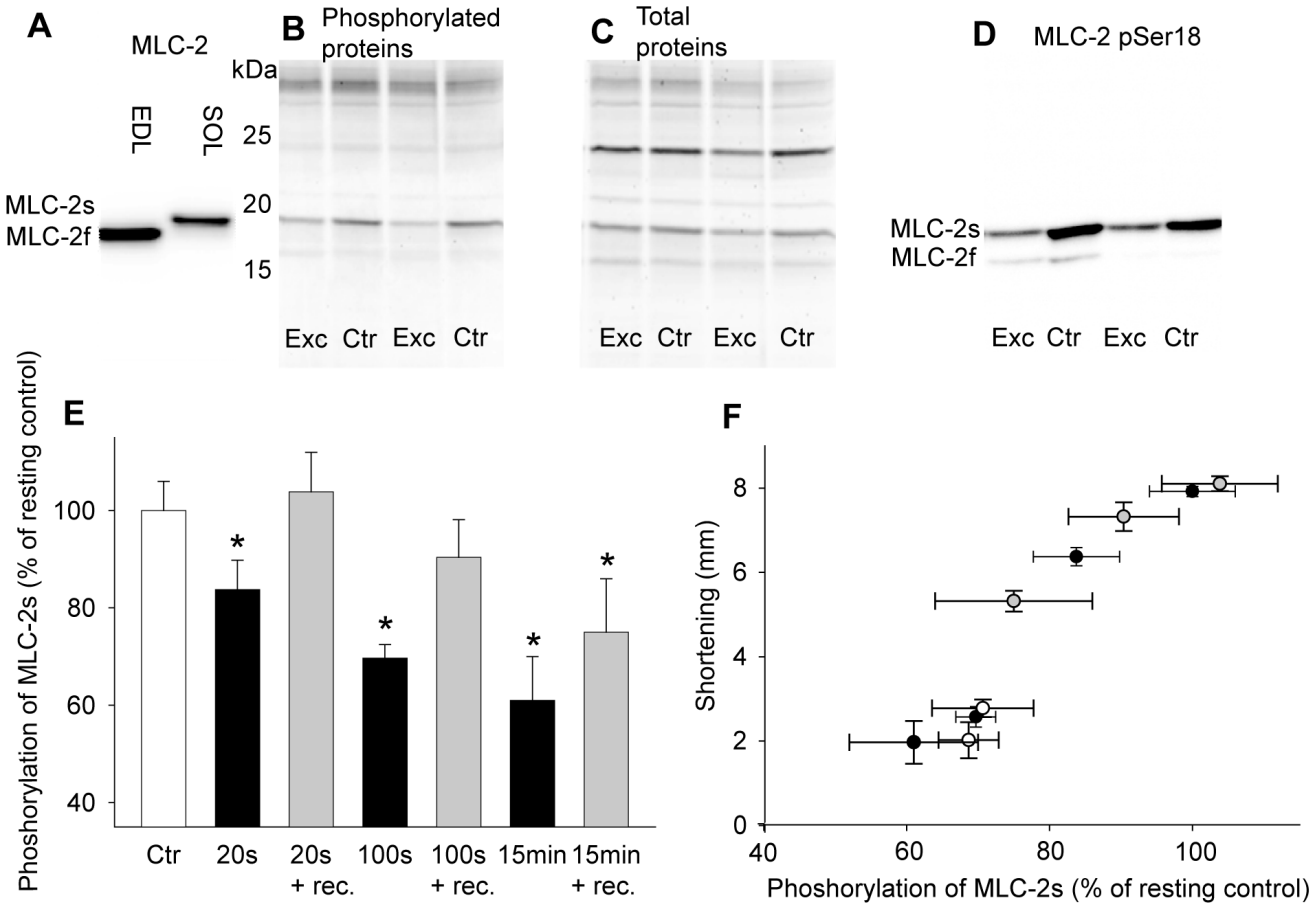
Dephosphorylation of MLC-2s. *A*) Immunoblot of the extensor digitorum longus (EDL) and soleus (SOL) muscle probed with monoclonal MLC-2 antibody. *B*) Gel showing myofibrillar proteins from SOL stained with ProQ Diamond for phosphorylated proteins. *C*) The same gel as in *B* stained with Sypro Ruby for total proteins. The phosphorylation level of MLC-2s was normalized to protein content of MLC-2s in individual muscles by dividing the staining intensity reflecting phosphoryration level of the MLC-2s (ProQ Diamond) by the staining intensity of the MLC-2s protein band (Sypro Ruby). This normalized phosphorylation level was then calculated relative to the resting, contralateral control muscle. *D*) Immunoblot of SOL probed with MLC-2 pSer18 confirmed the same phosphorylation pattern as seen with ProQ Diamond gel stain. *E*) MLC-2s phosphorylation in exercised SOL after different exercise durations (black bars) and after respective recovery periods (grey bars) in the 1^st^ bout, relative to the resting control muscle (100%, white bar). Bars are averages ± SEM. *F*) MLC-2s phosphorylation plotted against maximal shortening at all measured time points in the 1^st^ bout (black), after recovery (grey) and in the 2^nd^ bout (white). Symbols are group means ± SEM. *p<0.05 *vs.* control. *N,* 20 s = *6*; 20 s+ recovery = *7*; 100 s = *15*; 100 s+recovery = *6*; 15 min = *8*; 15 min+recovery = *6*. 100 s 2^nd^ bout = *6*; 15 min 2^nd^ bout = *7*.

Immunoblot analysis verified the band of the slow isoform of myosin light chain 2 (MLC-2s) (Fig. 8A). Gel analyses with sequentially staining of the gels with ProQ Diamond and Sypro Ruby for phosphorylated and total proteins, respectively (Fig. 8B–C), allowed normalization of MLC-2s phosphorylation relative to the protein content of MLC-2s. The normalized phosphorylation level was subsequently calculated relative to the contralateral resting control leg (100%). Immunoblot analysis with anti-MLC-2 pSer18 (Fig. 8D) confirmed the phosphorylation pattern observed with ProQ Diamond.

Neither preparation of the muscle nor the anaesthetic gases altered the phosphorylation level of MLC-2s in soleus (data not shown). The phosphorylation level of MLC-2s decreased with exercise time (Fig. 8E), and was significantly reduced already at 20 s (84±6%, p<0.05 vs. ctr) with a further decline at 100 s (70±3%, p<0.05 vs. ctr) and 15 min exercise (60±6%, p<0.05 vs. ctr), associated with simultaneous reduction in S_max_. The resting period following 20 s and 100 s exercise resulted in fully recovered phosphorylation level of MLC-2s (Fig. 8E), followed by fully recovered S_max_. However, following 15 min exercise, 15 min rest was not sufficient to fully recover the phosphorylation level of MLC-2s (75±11%, p<0.05 vs. ctr) (Fig. 8E), accompanied by only partial recovery of S_max_.

In the 2^nd^ bout, the phosphorylation level of MLC-2s was measured at start (i.e. after 15 min rest, se above) and after 100 s and 15 min exercise. The phosphorylation level was 71±7% after 100 s and 69±4% after 15 min, not different from the corresponding 1^st^ bout values of MLC-2s phosphorylation.

Interestingly, in contrast to metabolites, we found the phosphorylation level of MLC-2s to be correlated to S_max_ at all measured time points in the two exercise protocols as well as after recovery, shown in figure 8F.

### Additional Protocols with Analysis of the Phosphorylation Level of MLC-2s

**Figure 9:**
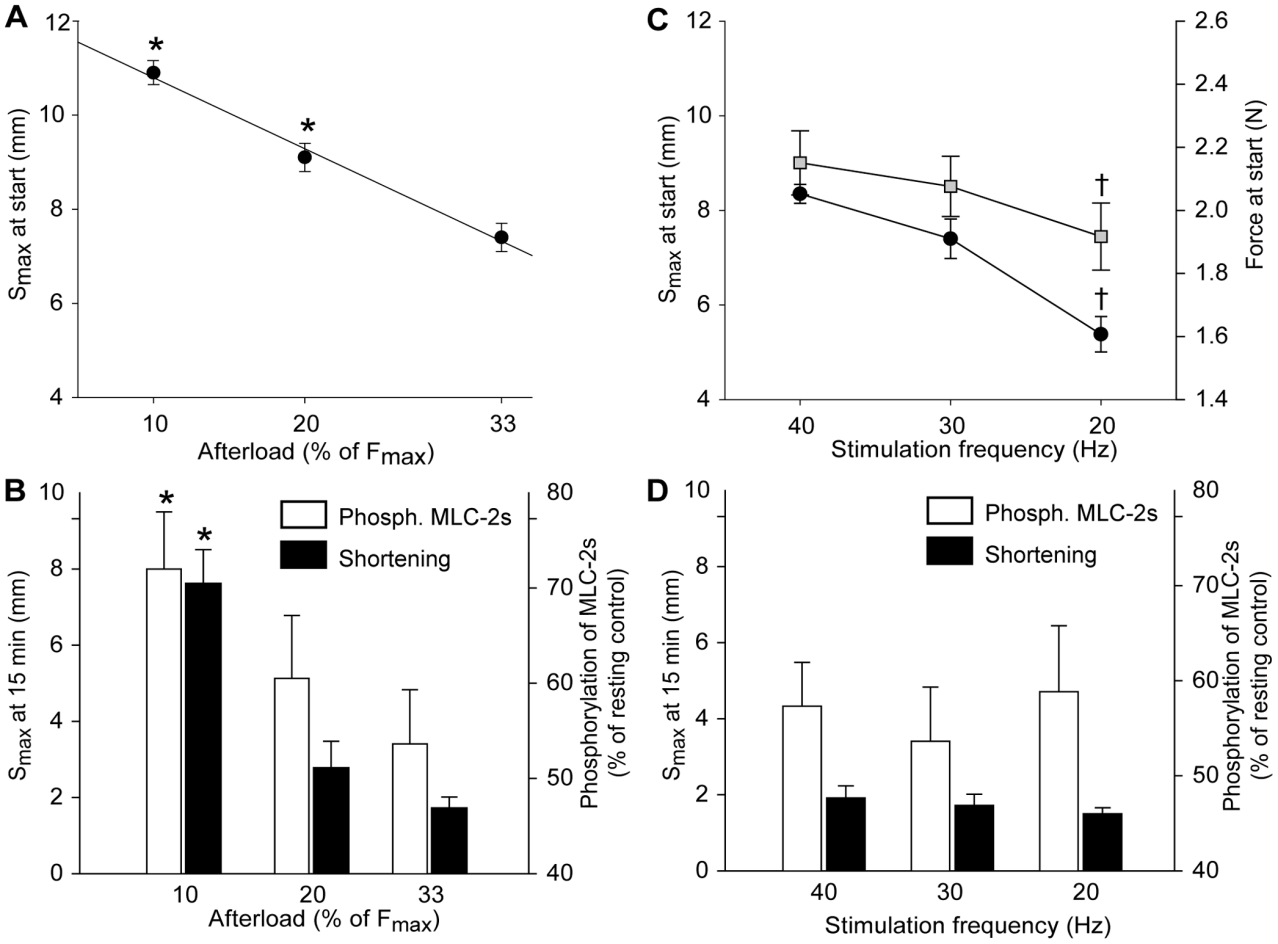
Effects of afterload and stimulation frequency on dephosphorylation of MLC-2s. Panel *A* and *B*: The effects of altered afterload of the exercising muscle. Stimulation frequency was 30 Hz as in the standard protocols. *A*) Shortening (S_max_) at start was strongly correlated to the pre-set afterload (r^2^ = 0.99). *B*) The phosphorylation level of MLC-2s (white bars) relative to resting control (100%) and the corresponding S_max_ (black bars) at 15 min exercise. Afterload was set to 10, 20 or 33% of F_max_. Panel *C* and *D:* The effects of altered muscle stimulation frequency. Afterload was 33% of F_max_ as in the standard protocols. *C*) S_max_ (black circles) and force development (grey squares) in the unfatigued muscle at various stimulation frequencies. *D*) The phosphorylation level of MLC-2s (white bars) and S_max_ (black bars) at 15 min exercise with stimulation frequency 40, 30 or 20 Hz. Symbols are averages ± SEM. *p<0.05 *vs.* 33% afterload. †p<0.05 *vs.* 30 Hz. *N* 10% afterload = *6*; 20% afterload = *5*; 33% afterload = *8*; 20 Hz = *6*; 40 Hz = *6*.

The effect of afterload on MLC-2s phosphorylation was investigated by performing supplementary 15 min exercise protocols with the load either decreased or increased compared to the default (33% of F_max_) load. Lowering the load to 20 and 10% of F_max_ revealed a linear, inverse relationship between load and S_max_ at start of the exercise protocol (Fig. 9A). At 15 min exercise S_max_ was less reduced in muscles that shortened against the smallest load, and the dephosphorylation of MLC-2s paralleled the reduction in S_max_ (Fig. 9B).

When the load was increased to 100% of F_max_, contractions were isometric. Notably, contrary to the protocols of shortening contractions; in this protocol of isometric contractions there was no dephosphorylation of MLC-2s at 15 min exercise (phosphorylation level 99±6% of resting control, data not shown).

To investigate whether improved preservation of S_max_ and phosphorylation level of MLC-2s could be attained by increasing the stimulation frequency, afterload was constant at the default 33% of F_max_ while stimulation frequency was either 20, 30 or 40 Hz. Force-frequency and length-frequency curves obtained before initiating the exercise protocol showed reduced maximal isometric force development and reduced S_max_ at lower stimulation frequencies, respectively (Fig. 9C). However, at 15 min exercise, S_max_ had stabilized at the same length in the 20, 30 and 40 Hz group, and the phosphorylation level of MLC-2s was not different between groups (Fig. 9 D).

### SR Calcium Handling

**Figure 10:**
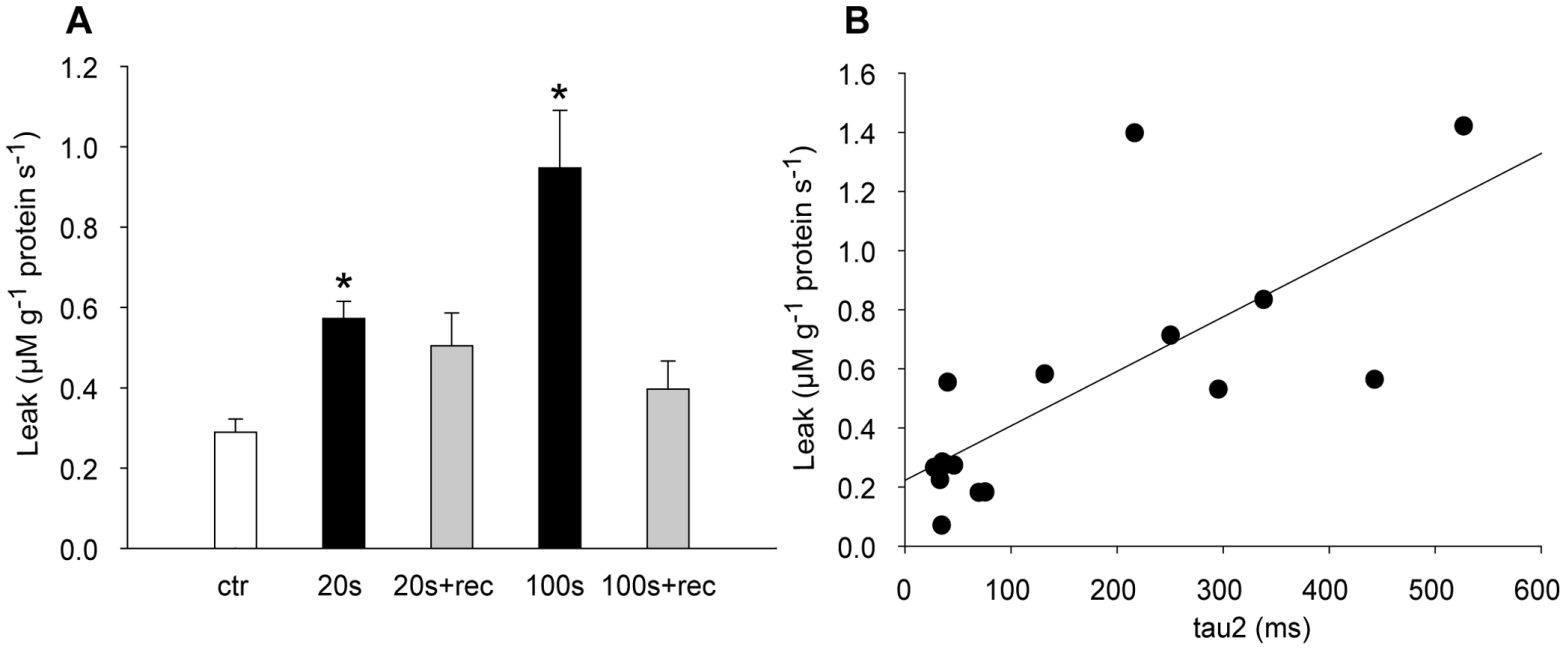
SR calcium leak. *A)* SR calcium leak after 20 s and 100 s exercise and after recovery from 20 s and 100 s exercise (2.5 min and 15 min recovery, respectively). *B)* Individual SR calcium leak values from start, 20 s and 100 s time points plotted against corresponding tau2 values (r^2^ = 0.55, p<0.01). Bars are means ± SEM (*N* Ctr = *8*; 20 s = *4*; 20+ rec = *4*; 100 s = *6*; 100 s+rec = *4*).*p<0.05 *vs.* control.

Sarcoplasmic reticulum (SR) Ca^2+^ handling was analysed after 20 s and 100 s of shortening contractions and at the end of the consecutive recovery periods (2.5 min and 15 min rest, respectively). SR leak was significantly increased after 20 s and 100 s exercise compared to control (Fig. 10A), and was not different from control after rest. The increase in SR leak correlated with the pronounced augmentation in tau2 from start to 100 s exercise (Fig. 10B). We did not detect any significant differences between groups in maximal rate of SR Ca^2+^ release or SR Ca^2+^ uptake (data not shown).

## Discussion

The present study demonstrates that repeated shortening contractions for 15 min (30 Hz, 1 s on/off) affect skeletal muscle contractile function in a complex pattern. We identified three phases of fatigue development during the exercise protocol (Fig. 2B) which was carried out on the rat soleus muscle *in situ* with maintained blood supply and at physiological temperature. The most striking finding was the pronounced and persistent decline of shortening velocity and S_max_ consistent with the findings of Munkvik *et al* [2], while the isometric relaxation rate was only transiently slowed. The observation that the various rates and velocities did not deteriorate or recover in parallel indicates that muscle shortening, S_max_, is regulated by other mechanisms than muscle relaxation.

The main findings can be summarized as follows: 1) the first time phase involved rapid dephosphorylation of MLC-2s paralleled by reduced rate of force development and shortening velocity with a reduced S_max._ Fatigue was clearly evident as reduction in S_max_ lasting throughout the exercise time. 2) MLC-2s dephosphorylation was well correlated to S_max_ both during fatigue development and during recovery. 3) There was a profound slowing of isometric relaxation in the second phase of fatigue (20–100 s) with a resulting increase in T_bl_ at 100 s. This slowing of isometric relaxation was almost absent in a 2^nd^ bout of exercise starting 15 min after cessation of the 1^st^ bout. In the two bouts of exercise the breakdown of high-energy phosphates was not different, whereas accumulation of lactate was less at 100 s in the 2^nd^ bout compared to at 100 s in the 1^st^ bout. In addition SR Ca^2+^ leak was increased after 100 s exercise in the 1^st^ bout and was correlated to the slowing of relaxation. 4) During the third phase of fatigue (100 s −15 min) the slowing of isometric relaxation was restored to initial values in parallel with normalization of high-energy phosphates and elimination of lactate. 5) Recovery was rapid and complete after 20 s and 100 s of exercise. After 15 min exercise some contractile parameters, most conspicuously S_max_, did not fully recover within 15 min rest. In spite of this, relaxation parameters were improved in a subsequent exercise bout.

### Dephosphorylation of Myosin Light Chain 2

An important aim of the present study was to further elucidate the role of MLC-2s in slow twitch skeletal muscle. The phosphorylation level of MLC-2s was gradually reduced during exercise by almost 50% after 15 min, and correlated to the reduction in S_max_. In contrast to the muscle content of high-energy phosphates and lactate, MLC-2s phosphorylation remained correlated to S_max_ throughout the 2^nd^ exercise bout (Fig. 8F).

Interestingly, shortening with a tiny load (10% of F_max_) caused only a small dephosphorylation of MLC-2s. One might therefore expect that isometric contractions (100% afterload) would cause maximal changes of the phosphorylation level. This was not the case. In fact during isometric contractions the MLC-2s phosphorylation was not significantly changed. Thus only the combination of load and shortening (i.e. work) triggered dephosphorylation. Therefore, this study strongly supports a regulatory role for MLC-2s in shortening contractions, and we propose that dephosphorylation of MLC-2s may be a cause of fatigue in slow twitch muscle in the sense that it will decelerate velocity of shortening and hence lead to reduced S_max_ (Fig. 8F). Reduced release of Ca^2+^ is found to be an important cause of reduced force and F_max_ during isometric contractions [13], and we cannot rule out that reduced release of Ca^2+^ influence the velocity of shortening. However, both the reduction of S_max_ and MLC-2s dephosphorylation after 15 min exercise were independent of the stimulation frequency (Fig. 9D), indicating that Ca^2+^ is not a major cause of the reduction in shortening velocity.

It has been shown that MLC-2 dephosphorylation does not affect velocity of *unloaded* shortening [26] which was corroborated in the study of Munkvik *et al.* [2]. However, the number of attached cross-bridges is thought to be reduced by dephosphorylation [27], [28], which can explain that velocity of *loaded* shortening will be slower. Also the rate of isometric force development will be affected which fits with the present data, since changes of isometric force development closely followed the changes of velocity of shortening albeit not as pronounced. At 100 s additional factors may contribute to the slowing as discussed below. The proposed role of MLC-2s is supported by data from cardiac muscle, since transgenic mice expressing a non-phosphorylatable MLC-2 demonstrated depression of load-dependent contractility [29], and manipulating the phosphorylation of MLC-2 to a lower level in cardiac trabeculae produced reduced isometric force, shortening velocity and generated power [30]. These effects of dephosphorylation seem to be partly reflected in an apparent right-shift in the force-pCa curve [12].

Such a role of the MLC-2s isoform in slow twitch muscle is in sharp contrast to the role of the MLC-2f isoform in fast twitch muscle since for the latter several studies have shown that muscle activity leads to hyperphosphorylation and post-tetanic twitch potentiation [for review see 11]. The study of Munkvik *et al.* [2] and the present study, both from our laboratory, are the first to unequivocally show that shortening contractions under physiological conditions in slow twitch muscle lead to rapid dephosphorylation of MLC-2s. This indicates that the phosphorylation level is high in the resting state.

The different role of MLC-2 in slow twitch compared to fast twitch fibres may partly be due to different isoforms, but clearly also to different pathways for phosphorylation and dephosphorylation. In fast-twitch muscle, the myosin light chain kinase (skMLCK) is activated by Ca^2+^/calmodulin, probably explaining why muscle activity leads to hyperphosphorylation (for review see [11]). Dephosphorylation is carried out by protein phosphatase type 1 associated with a regulatory subunit [31]. Therefore, our data indicate that Ca^2+^ activation of skMLCK is not a major regulator of the activity-dependent phosphorylation level of MLC-2s in slow twitch muscle. Dephosphorylation must either be due to downregulation of kinase activity or activation of a phosphatase. To our knowledge there is very little information about these pathways in slow-twitch muscle. Reports of different ratios of MLC-kinase and phosphatase in slow twitch and fast twitch [32] as well as frequency-dependent phosphorylation of MLC-2 in fast twitch but not in slow twitch [12] support the presumption that the regulation of MLC-2 is dissimilar in fast- and slow twitch muscle. We cannot rule out that in slow twitch muscle Ca^2+^ could play a role in activating a phosphatase. Alternatively, mechanical performance may be sensed by the cell. The giant molecule titin has regions that are sensitive to stress and strain and can function as a mechanotransducer that triggers intracellular signals (for review see [33]). However, the link to mechanical performance is not straightforward since dephosphorylation seems to be associated with afterload, shortening and duration of exercise. We found that MLC-2s was rapidly re-phosphorylated during recovery from 20 s exercise, and at least part of the re-phosphorylation was much slower after 100 s and 15 min exercise, possibly explaining the relatively slow recovery of S_max_. In fact, neither S_max_ nor phosphorylation level of MLC-2s was back to normal after 15 min rest following the 1^st^ exercise bout. Hence, there seems to be two components of MLC-2s dephosphorylation and re-phosphorylation and that the slow component only becomes apparent when exercise duration exceeds 100 s.

### Metabolic Correlates of Fatigue

The pronounced slowing of relaxation with the resulting rise in T_bl_ was characteristic for the second phase of fatigue (20–100 s exercise in the 1^st^ bout). Slowing of relaxation is a hallmark of skeletal muscle fatigue and can limit performance; during dynamic exercise with alternating movement (e.g. running), slowing of relaxation will probably be experienced by the individual as muscle stiffness. Due to the slowing of relaxation, the muscle is no longer capable of keeping up the same frequency. If the frequency (for instance the running pace) is to be maintained, the antagonist muscle has to employ force to stretch out the limb in between contractions of the agonist muscle.

The slowing of isometric relaxation at 100 s was paralleled by a many-fold increase in lactate. An important finding of this study was that isometric relaxation rate was strongly correlated to lactate throughout the exercise protocols (Fig. 7), but less well correlated to high-energy phosphate levels and degree of MLC-2s phosphorylation. Notably, the pronounced rise in T_bl_ seen at 100 s in the 1^st^ bout was almost undetectable in the 2^nd^ bout, corresponding to significantly less accumulation of lactate.

We have previously reported the metabolite content at 15 min exercise in the 1^st^ bout to involve full restoration of CrP and partial restoration of ATP and lactate [2], and in the present study a similar restoration of metabolites was found in the 2^nd^ bout (Table 2). Hence, we propose that the restoration of metabolites contributes to the observed partial restoration of isometric contractile function at 15 min exercise, both in the 1^st^ and the 2^nd^ bout. However, S_max_ and hence work performed was not restored. It seems at 15 min that work performed was stabilized at a reduced level that corresponded to an energy requirement not exceeding the aerobic metabolic capacity of the muscle. This is in contrast to the first 100 s of exercise during which contractile performance clearly also triggered a large anaerobic ATP production.

Muscles fatigue more rapidly during shortening contractions compared to isometric contractions [3]–[6], possibly explained by a higher rate of ATP consumption during shortening contractions [34]. Above the anaerobic threshold inorganic phosphate (P_i_) will increase, which has been suggested to be a major cause of fatigue during shortening contractions [6]. The considerable breakdown of high-energy phosphates seen after 100 s in the 1^st^ bout would be expected to produce such an increase in P_i_. Accumulation of P_i_ may decrease myofibrillar Ca^2+^-sensitivity, diminish myofibrillar force production due to inhibition of the transition to the high-force generating cross-bridge state (the release of P_i_) and possibly reduce Ca^2+^ release from the SR due to Ca^2+^-P_i_ precipitation (for review see [35]). Hence, breakdown of high-energy phosphates and accumulation of P_i_ are likely to have an effect on isometric force development in the present experiments.

The release of P_i_ [9] or the release of ADP [36] are considered to be rate limiting steps in the cross-bridge ATPase cycle. Increased ADP has been reported to depress shortening velocity, but possibly increase force [37], concievably by hampering cross-bridge detachement [38]. However, ADP did not seem to play a major role in the present experiments, since no significant changes were found throughout the exercise protocol.

We found that the isotonic relengthening velocity was very sensitive to exercise (Fig. 6D), displaying a slowing that was most pronounced at the marked metabolic depletion at 100 s exercise. However, isotonic relengthening velocity was only partially restored at 15 min exercise (contrary to isometric relaxation rate witch was fully restored (Fig. 6E) in parallel with metabolites), but because of the persistent reduction in S_max_ (Fig. 6C) the relengthening time (TTL_0_) was fully restored. The possibility remains that dephosphorylation of MLC-2s could contribute to slowing of isotonic relengthening velocity as well as shortening velocity by reducing the number of attached cross-bridges. The literature is sparse regarding factors governing the isotonic relengthening phase, and further studies are needed to elucidate the mechanisms at play.

We confirm that depletion of high-energy metabolites plays an important role in fatigue development during shortening contractions, however, metabolites alone (including lactate) are inadequate to explain all the contractile changes. In agreement with several reports [39]–[42] we conclude that acidosis caused by lactate accumulation is closely associated with slowing of isometric relaxation. The slowing could be mediated through a direct effect on the cross-bridges, or through a reduced rate of removal of Ca^2+^ from the myoplasm (see below).

### SR Calcium Handling

A slow rate of SR Ca^2+^ reuptake by the SR accompanied by a slow dissociation of Ca^2+^ from TnC could contribute to a decrease of the isometric relaxation rate. The effect of lactacidosis on the intracellular Ca^2+^-handling is controversial. However, there is evidence suggesting that acidification reduces the rate of Ca^2+^ reuptake by SERCA [39], [41], providing a possible explanation for the observed correlation between lactate and isometric relaxation rate in the present study (Fig. 7). Under optimal metabolic conditions we did not detect reduced SR Ca^2+^ uptake in SR vesicles isolated at 100 s exercise. However, the vesicle studies were performed at normal pH which would preclude detection of the *in vivo* effect of the lactacidosis. This is in accordance with the findings of Ørtenblad *et al* [43] who reported slowing of relaxation without alterations in *in vitro* SR Ca^2+^ uptake rates.

Interestingly, there was a significant increase in vesicle SR Ca^2+^ leak at 100 s exercise highly correlated to the prolongation of the time constant tau2 of the isometric relaxation (Fig. 10B). This finding could suggest that leaky ryanodine receptors contributed to the transient slowing of isometric relaxation. A continuous leak from SR would cause a futile cycling of Ca^2+^ and increase the demand on SERCA. Consequently, the decay of the cytosolic Ca^2+^ transient would be slower. In general, exercise is associated with activation of the sympathetic nervous system. In skeletal muscle, ß-adrenergic stimulation has been reported to lead to activation of protein kinase A and subsequent phosphorylation of skeletal muscle proteins including the ryanodine receptor 1 (RyR1), which on phosphorylation releases the stabilizing protein calstabin 1 (also called FKBP12) resulting in increased leak through the channel [14]. Although in that study ß-adrenergic activity was chronically elevated, our results suggest that increased Ca^2+^ leak could play a role also in acute fatigue development.

In isometric contractions, reduced SR Ca^2+^ release is considered to be an important contributor to fatigue after prolonged stimulation, presumably due to precipitation of Ca^2+^ by P_i_ in the SR (for review see [13]). Since in the present experiments fatigue was fully developed at 100 s, any contribution of reduced Ca^2+^ release should have occurred at this time point. In the vesicle studies we did not detect a reduction of SR Ca^2+^ release at 100 s. This does not preclude that reduced Ca^2+^ release from the SR was present *in vivo*. However, the modest reduction of F_max_ observed by Munkvik *et al.* [2] at 100 s does not support a significant contribution of reduced Ca^2+^ release.

The incomplete recovery of isometric force development at start of the 2^nd^ bout could possibly also be sought in SR Ca^2+^ handling. It is believed that prolonged exercise can produce a delayed recovery of SR Ca^2+^ release, reported to reduce force especially at low stimulation frequencies, and neither metabolites nor action potential changes seem to provide an explanation to this type of SR Ca^2+^ dysfunction [44]. The cause could reside in some kind of structural change in the ryanodine receptor or its interacting regulators, however the mechanism remains uncertain. We cannot exclude that the findings of Edwards *et al.* applies also when employing shortening contractions. Further, the delayed recovery could reside in altered myofibrillar Ca^2+^ sensitivity due to ROS-induced modifications [45], and future studies are needed to sort out the contribution of altered intracellular Ca^2+^ handling in shortening contractions, as many questions remain unsolved.

In summary with regard to Ca^2+^ handling, the close relationship between isometric relaxation and lactate could possibly be due to acidification-induced slowing of Ca^2+^ reuptake by SERCA. Further, slowing of isometric relaxation seemed to correlate well with increased Ca^2+^ leak from the SR. As for the other parameters of muscle fatigue, a contribution of altered intracellular Ca^2+^ handling cannot be ruled out, but seems not to be a major factor.

### Improved Oxidative Metabolism

In spite of only partial recovery of contractile parameters at the start of the 2^nd^ bout, fatigue at 100 s exercise in the 2^nd^ bout was less than at 100 s in the 1^st^ bout. Activation of oxidative metabolism occurs more slowly than the rapid activation of non-oxidative ATP delivery during the initial phase of muscle activity, and it remains to fully map whether the delay is due to an inertia of the intrinsic cellular metabolic activation or of the extrinsic oxygen transport mechanisms, or possibly both (for review see [46]). Munkvik *et al.* [2] reported normal tissue oxygen levels at the 100 s time point in the present *in situ* exercise protocol, which could point to an inertia of intracellular processes. A priming bout of exercise at an intensity exceeding the lactate threshold has been reported to speed up oxygen uptake (VO_2_) on-kinetics [17]–[19], and the less accumulation of lactate observed in the 2^nd^ bout in our study is in accordance with these findings. Hernández *et al.* [47] recently investigated VO_2_ and blood flow kinetics in a canine *in situ* model. They found that a prior bout of isometric contractions speeded up the primary time constant and reduced the VO_2_ slow-component amplitude in the second bout, in concert with an improved adjustment of convective O_2_ delivery. The possibility remains that similar changes take place also in the present *in situ* model of shortening contractions. Interestingly, the study of Hernández *et al*. [47] agrees with our finding of reduced maximal contractile performance at start of the second bout, in spite of an improved oxidative ATP-delivery.

### Conclusion

In an *in situ* exercise protocol of shortening contractions, fatigue developed in three distinct phases. There was an early and rapid dephosphorylation of MLC-2s which we propose is linked to the decline of isotonic shortening velocity through a slowing of cross-bridge turnover rate. With the present protocol the most striking consequence of reduced isotonic shortening velocity was the reduction of S_max_ (and hence work) which started early and then stabilized at a level where the energy demand of the muscle could be met by aerobic metabolism. The initial high work load, before S_max_ was reduced to its stable level, taxed the anaerobic metabolism so that high-energy phosphates were reduced and lactate accumulated. These changes were conspicuously related to slowing of muscle relaxation which was compromised in the second time phase, culminating at 100 s exercise. Slowing of isometric relaxation was closely associated with lactate accumulation and with increased leak of Ca^2+^ from the SR. During the third phase, the work performed was sufficiently low to allow for restoration of high-energy phosphates and partial restoration of lactate. Thus, effects on relaxation disappeared, however low phosphorylation level of MLC-2s and reduced S_max_ prevailed. The phosphorylation level of MLC-2s was influenced by working load, but not by stimulation frequency. Altogether, fatigue is a complex phenomenon, highly related to the metabolic demand of the muscle, and we propose that the regulatory protein MLC-2s participate in regulating the capacity to shorten and perform work.
